# The microbiome of captive hamadryas baboons

**DOI:** 10.1186/s42523-020-00040-w

**Published:** 2020-07-16

**Authors:** Xuanji Li, Urvish Trivedi, Asker Daniel Brejnrod, Gisle Vestergaard, Martin Steen Mortensen, Mads Frost Bertelsen, Søren Johannes Sørensen

**Affiliations:** 1 Section of Microbiology, Department of Biology, University of Copenhagen, 2100 Copenhagen, Denmark; 2 Novo Nordisk Foundation Center for Basic Metabolic Research, Section of Metabolic Genetics, University of Copenhagen, Copenhagen, Denmark; 3 Center for Zoo and Wild Animal Health, Copenhagen Zoo, 2000 Frederiksberg, Denmark

**Keywords:** Baboon, Group living, Body habitat, Microbiota, Beneficial bacteria, Health

## Abstract

**Background:**

The hamadryas baboon (*Papio hamadryas*) is a highly social primate that lives in complex multilevel societies exhibiting a wide range of group behaviors akin to humans. In contrast to the widely studied human microbiome, there is a paucity of information on the host-associated microbiomes of nonhuman primates (NHPs). Here, our goal was to understand the microbial composition throughout different body sites of cohabiting baboons.

**Results:**

We analyzed 170 oral, oropharyngeal, cervical, uterine, vaginal, nasal and rectal samples from 16 hamadryas baboons via 16S rRNA gene sequencing. Additionally, raw Miseq sequencing data from 1041 comparable publicly available samples from the human oral cavity, gut and vagina were reanalyzed using the same pipeline. We compared the baboon and human microbiome of the oral cavity, gut and vagina, showing that the baboon microbiome is distinct from the human. Baboon cohabitants share similar microbial profiles in their cervix, uterus, vagina, and gut. The oral cavity, gut and vagina shared more bacterial amplicon sequence variants (ASVs) in group living baboons than in humans. The shared ASVs had significantly positive correlations between most body sites, suggesting a potential bacterial exchange throughout the body. No significant differences in gut microbiome composition were detected within the maternity line and between maternity lines, suggesting that the offspring gut microbiota is shaped primarily through bacterial exchange among cohabitants. Finally, *Lactobacillus* was not so predominant in baboon vagina as in the human vagina but was the most abundant genus in the baboon gut.

**Conclusions:**

This study is the first to provide comprehensive analyses of the baboon microbiota across different body sites. We contrast this to human body sites and find substantially different microbiomes. This group of cohabitating baboons generally showed higher microbial diversity and remarkable similarities between body sites than were observed in humans. These data and findings from one group of baboons can form the basis of future microbiome studies in baboons and be used as a reference in research where the microbiome is expected to impact human modeling with baboons.

## Background

Humans and other primates are home to trillions of symbiotic microorganisms. Interactions between a host and its microbes affect host physiology, behavior, reproduction, immunity and evolution [1–3]. The Human Microbiome Project, through monitoring or manipulations of the human microbiome, helps us better understand the associations between microbes and human health [4]. In contrast to the widely studied human microbiome, there is a paucity of information on the host-associated microbiomes of nonhuman primates (NHPs). Information about NHP microbiota is essential for understanding the factors underlying microbial coevolution with their hosts [5, 6]. Broad primate microbiome surveys could also allow for the development of predictive biomarkers to improve nonhuman primate health and management.

Baboons (genus *Papio*) are one of the most biologically relevant research animal models due to their genetic and physiological similarities to humans [7]. Baboons are large-bodied, omnivorous, highly social, terrestrial Old World African monkeys that occupy a wide array of habitats similar to those of early hominins [8, 9]. Of the six recognized species [10], the social system of hamadryas baboons shares more similarities with humans than that of other baboons [9]. Like modern humans, the hierarchical social networks of hamadryas baboons connect individuals at multiple levels [8]. Frequent social interactions (mostly grooming) are necessary for baboons to maintain affiliative bonds [11].

To our knowledge, only the rectal and vaginal microbiota of baboons have been examined, likely because baboons can be used as a model in gastrointestinal and female reproductive studies due to the features shared with humans [12–14]. In this study, we investigated 170 samples of rectal, oral, oropharyngeal, cervical, uterine, vaginal and nasal microbiota from 16 captive hamadryas baboons by culture-independent sequencing of the 16S rRNA gene hypervariable V3-V4 region. Our study provides detailed insights into the baboon microbiome structure and ecology.

## Results

### Microbial distribution in different body sites of captive baboons

**Fig. 1:**
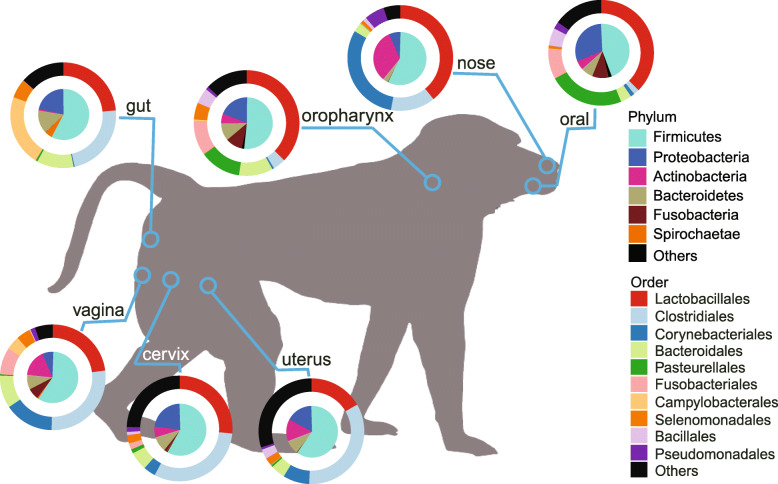
Microbial composition on phylum and order levels of each anatomic site. Pie charts show the average microbial composition of seven body habitats on order (donut chart) and phylum level (pie chart), respectively. Lactobacillales were found in all the body sites (Mean 27% ± SD 10%)

Six main phyla were detected in the seven body sites of 16 captive hamadryas baboons (*Papio hamadryas,* 13 females, 3 males). The phylum Spirochaetae was found to be abundant in the baboon gut (Fig. 1). Firmicutes dominated in all body sites. The dominance of other phyla varied among body sites; for instance, Fusobacteria was dominant in the oral cavity and oropharynx, while Actinobacteria predominated in the nose. On the order level, we found Lactobacillales to be abundant in all seven body sites (Mean 27% ± SD 10%), especially in the oral cavity, oropharynx and nose, constituting around 40% of the total bacteria. In addition to Lactobacillales, Clostridiales were predominant in the vagina, cervix, uterus, and gut, accounting for 16–27% of the microbiota. The oral cavity and oropharynx shared a microbial profile mainly composed of Lactobacillales, Bacteroidales, and Pasteurellales, representing over 50% of the microbiota. Lactobacillales and Corynebacteriales were the two major bacteria in the nose, together comprising almost 70% of the microbiota.

### Microbial characterization varied among the body sites

**Fig. 2:**
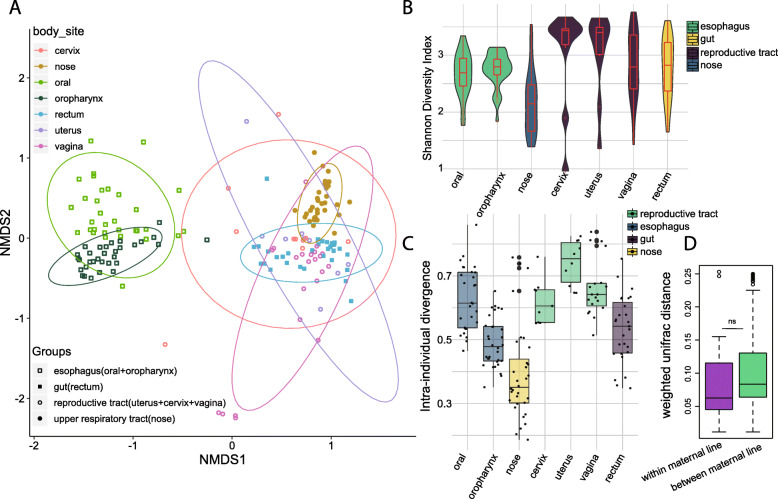
Microbial characterization in different body sites. **a** Non-metric multidimensional scaling (NMDS) based on Bray-Curtis distance of microbial communities from the baboon oral cavity, oropharynx, nose, cervix, uterus, vagina and rectum. The colored lines surrounding each sample type are covariance ellipsoids. **b** Alpha diversity in different body habitats, grouped by area, as measured using the Shannon index of ASV-level bacteria. Pharynx and nose had a significantly lower diverse microbiota than reproductive tract (TukeyHSD). **c** Divergence of microbes in a specific body site was quantified as the average dissimilarity of each sample from the group mean. **d** Weighted Unifrac distance within and between maternal lines for gut microbiota. ns means no significant difference detected between the same maternal line and different maternal lines by t-test (*p* = 0.19)

To investigate microbial features of different body sites, we analyzed microbial diversity between and within the different body sites, quantified the microbiome divergence within each body site, and compared gut microbial diversity between and within maternal lines (Fig. 2). Permutational Multivariate Analysis of Variance (PERMANOVA) using Bray-Curtis distances, visualized by Non-metric multidimensional scaling (NMDS) (Fig. 2a) showed that the oral cavity, oropharynx, and nose had unique microbial profiles but the microbial profiles in the cervix, uterus, vagina, and rectum did not have significant differences (pairwise comparison, adjusted *p* values are listed in Table S1). The same NMDS plot but showing the host source is shown in Supplementary Fig. 1. Microbial alpha-diversity in the baboon nose was significantly lower than in the other body sites (adjusted *p* values are listed in Table S2, TukeyHSD) (Fig. 2b). The divergence of microbiomes within each body site was quantified and extrapolated (Fig. 2c). The microbiomes in baboon nose and oropharynx had a smaller dispersion but were more heterogeneous in the reproductive tract and oral cavity (adjusted *p* values are listed in Table S3, Pairwise Wilcoxon Rank Sum Tests). The vertical inheritance of gut microbial communities was analyzed by comparing the weighted Unifrac distances between microbiomes of the same maternal line and microbiomes of different maternal lines (Fig. 2d). Statistical analysis showed no significant difference in weighted Unifrac distance within the maternal lines compared to between the maternal lines (t-test, *p* = 0.194), indicating the vital role of horizontal exchange in shaping the gut microbiota.

### Baboons had significantly different vaginal, gut and oral microbiomes from humans

**Fig. 3:**
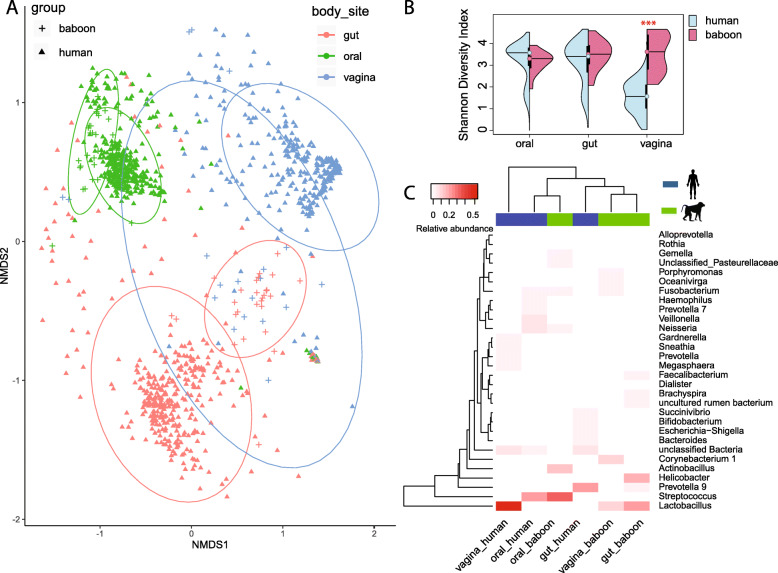
Comparisons between the human and baboon microbiomes in the oral cavity, gut and vagina. **a** Non-metric multidimensional scaling (NMDS) based on a Bray-Curtis distance matrix of microbial communities from baboon and human oral, gut and vagina. The colored lines surrounding each sample type are covariance ellipsoids. **b** Microbial alpha diversity in the human and baboon gut, oral cavity and vagina, as measured using the Shannon index of ASV-level bacteria. **c** Heatmap showing the 30 most abundant bacterial genera in human and baboon oral, nose and vagina

The sequencing data from 1041 human oral cavity, gut and vaginal samples (EBI databases available through study ID PRJEB14941) [15], processed using similar DNA extraction, DNA kit and sequencing platform, were analyzed using the same pipeline as used for the baboon. To ensure that the sample types were comparable between baboons and humans, we compared the homogeneity of dispersions for baboons and humans for each sample type. The average dispersion within sample types from baboons and human did not show significant differences (baboon gut: 0.4, human gut: 0.5, baboon oral: 0.3, human oral: 0.37, baboon vagina: 0.5, human vagina: 0.43, adjusted *p* value > 0.5, adjusted *p* values for all pairwise comparisons are shown in Supplementary Table S4). We found that the baboon microbiomes in the three body sites were significantly different from human microbiomes (pairwise comparison, adjusted *p* = 0.015 for oral vs oral microbiome, gut vs gut microbiome and vaginal vs vaginal microbiome, PERMANOVA, Fig. 3a).

The baboon vaginal microbiome had a higher alpha diversity than that of the human (adjusted *p* value <1e-7, TukeyHSD, Fig. 3b). The oral microbiome had a slightly higher alpha diversity in humans than in the baboon, while the gut microbiome showed similar alpha diversity in the two hosts. *Lactobacillus* (13% ± 12%) was not dominant in the baboon vagina, as it is in the human vagina (54% ± 37%), but it was the most abundant genus in the baboon gut (22% ± 17%) (Fig. 3c). In contrast, the human gut only contained a mean relative abundance of 0.35% *Lactobacillus* (Fig. 3c). Despite an overall significant difference in oral microbiome profiles between baboons and humans (Fig. 3a), they clustered together based on the abundances of the 30 most abundant genera overall (Fig. 3c). *Streptococcus* was the most abundant genus both in the human and baboon oral cavity.

### The microbiomes of group living baboons shared more similarities across body sites than in humans

**Fig. 4:**
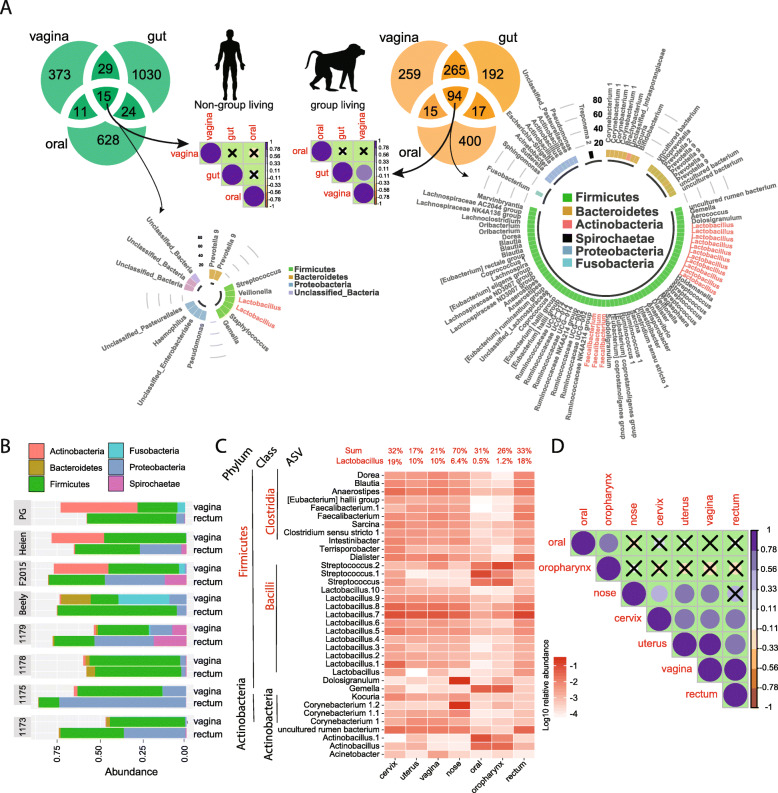
Shared microbial ASVs and their correlation analysis among human/baboon oral, gut and vagina, and among all seven body sites of baboons. **a** Venn diagrams showing the number of shared ASVs and spearman correlation matrix for shared ASVs among oral, gut and vagina of humans and baboons. X in the correlation matrix means no significant correlations. All the significant correlations shown in the matrix have an adjusted *p*-value< 0.01. Color intensity and the size of the circle are proportional to the correlation coefficients. The shared ASVs by human and baboon samples were plotted in the circular barplots. **b** Bar plots showing the 40 most abundant bacteria ASVs of vagina and rectum in eight baboons. **c** Heatmap for 35 ASVs shared by all body sites. The ASVs belonging to genus *Lactobacillus* and *Faecalibacterium* are marked in red. The relative abundances of *Lactobacillus* and the 35 ASVs are listed. **d** Spearman correlation analysis for 35 ASVs shared by seven body sites. Only the Spearman correlation coefficients (PCC) with an adjusted *p* < 0.01 were plotted. X in the correlation matrix means no significant correlations. Color intensity and the size of the circle are proportional to the correlation coefficients

Bacterial exchange during group living is inevitable. We define shared ASVs as those that were present in at least two or more of the body sites sampled. The different body sites of group living baboons shared more ASVs than those of humans (Fig. 4a). The abundances of these shared ASVs were not significantly correlated between the body sites in humans but had a significant positive correlation between gut and vagina in the baboons (Fig. 4a), indicating potential bacterial exchange. Of the 15 ASVs shared between the human oral cavity, gut and vagina, 5 ASVs belonged to unclassified taxa (Fig. 4a). Most of the 94 ASVs shared by the baboon’s oral cavity, gut and vagina belonged to Firmicutes (Fig. 4a). In addition, the human oral cavity, gut and vagina had unique microbial compositions as shown in Non-metric multidimensional scaling (NMDS) plot (pairwise comparison, adjusted *p* = 0.003 for oral vs gut microbiome, oral vs vaginal microbiome and gut vs vaginal microbiome, PERMANOVA, Fig. 3a). However, the baboon gut microbiome was similar to the vaginal microbiome (Fig. 2a) despite a large variance within a single individual (Fig. 4b). From Fig. 2a, it is clear that multiple body sites of baboons had similar microbial profiles. Considering the potential bacterial exchange across body sites among co-habitants, we analyzed the ASVs (Fig. 4c) shared by the 7 body sites and their correlations (Fig. 4d). We identified 35 shared ASVs representing 33% (17–70% in each body habitat) of the mean relative abundance (Fig. 4c) and the majority belonged to the phylum Firmicutes (Fig. 4c). Among these 35 shared ASVs, 11 ASVs belong to the genus *Lactobacillus* and 2 ASVs belong to the genus *Faecalibacterium*. The relative abundance of these 35 shared ASVs was positively correlated between cervix, uterus, vagina and rectum, between nose, cervix, uterus, and vagina, as well as between oral and oropharynx.

## Discussion

In our study, we found that the baboon microbiome has unique characteristics compared to the human microbiome. The microbiomes of group living baboons shared more similarities between body sites than observed in humans. A significant positive correlation of the relative abundance of shared ASVs among the different body sites suggested potential bacterial exchange throughout the body. We note that the baboons investigated in our study lived in the same environment and shared the same diet, and acknowledge that both diet [16] and to a lesser extent genetics [17, 18] are known to be important factors in shaping the human gut microbiome, and may have contributed towards the high similarity of baboon microbiomes found here.

Group living generally entails frequent social interactions, especially for highly social baboons [19], who use them to maintain affiliative bonds [11]. Bacterial exchange in a shared environment has previously been reported. Members of a shared environment have more similar gut and skin bacterial communities than individuals living separately, indicating that a shared lifestyle or environment affects the microbiome composition [5, 6, 18, 20]. In a study of the gut microbiota in wild chimpanzees, group living chimpanzees shared more of their gut microbiome than individuals from different groups, and dietary convergence did not explain the convergence among chimpanzee-gut microbiome community memberships during periods of high sociability [6]. In the study by Tung et al., the yellow baboon (*Papio cynocephalus*) gut microbiome was detected to have a high group specificity as well [5]. They found that even if two social groups of wild baboons shared almost the same diet, the gut microbiota of these two groups was still significantly different and shaped by the social interactions [5]. Therefore, we speculate that bacterial exchange was an important reason for microbial similarities between multiple body sites of the group living baboons investigated here. In addition, the gut microbiota within the three maternal lines did not show a significant difference in comparison to those of unrelated individuals, which indicated that the gut microbiota is more affected by the bacterial exchange between group members in general than transfer from mother to offspring. Our findings are consistent with the results previously reported [6]: inheritance of microbial communities across generations were primarily driven by horizontal transfer among interacting hosts. In our study, approximately one-third of all the shared ASVs were from the genera *Lactobacillus* and *Faecalibacterium*, indicating a higher tendency for these genera to be shared between body sites. Many species of *Lactobacillus* and *Faecalibacterium* are widely considered to be probiotics [21, 22], suggesting sociality may also foster commensal and mutualistic microbial diversity, which could potentially help explain the driving force for bacterial transmission.

In this study, we found that the baboon vaginal microbiome was similar to the gut microbiome, which is different from what has been seen in humans. For baboons, some grooming bouts, especially those directed from adult males towards estrous females, concentrate heavily on the anogenital region, increasing the probability of fecal-vagina transfer [5]. Besides, we found that the baboon vagina had a distinct microbiota profile from that of humans (Fig. 3a). Compared to humans, the polygynous mating system and promiscuity of the baboons probably boosted genital bacterial transfer among group members. The human vaginal samples analyzed in our study were from pregnant women and some were given a yogurt supplement [15]. There are differences in the composition and stability of the vaginal microbiota between pregnant and non-pregnant women [23], which can influence the comparison between human gut and vaginal microbial compositions. Nevertheless, normal healthy humans are known to have completely different gastrointestinal and urogenital microbiomes [24], which is different from what we find in the baboons. The baboon vagina had a relatively high microbial diversity (Fig. 3b) and *Lactobacillus* was not as predominant in the baboon vagina as in humans (Fig. 3c). Similar findings have been reported in other studies [13]. The human vagina is primarily colonized by *Lactobacillus* [25], which maintains an acidic environment and prevents the invasion of nonindigenous strains and potential pathogens and can account for 65.9 to 98.1% of the vaginal microbiota [26–29]. However, to some extent, the baboon vaginal microbial profiles were characterized by low *Lactobacillus* abundance, low lactic acid concentration and a higher, near-neutral vaginal pH [30], which are typically associated with bacterial vaginosis in the human vagina [31].

The baboon oral cavity had a unique microbial distribution and exhibited only minor overlap with the other body sites (Fig. 2a); it had somewhat lower microbial diversity than humans (Fig. 3b), despite the fact that humans exhibit more oral hygiene practices. Cleaner teeth could be argued to lead to higher diversity, as no bacteria will be allowed to grow to dominate the oral cavity, enabling a higher diversity of bacteria to be present at any given moment. The baboon gut microbiome is unique compared to the human gut microbiome. The baboon gut had high relative abundances of Lactobacillales and Clostridiales, which was consistent with previous findings [32]. In this previous study, the gut microbiome of captive olive baboons (*Papio Anubis*) was also reported to be significantly different from human gut microbiome. In our study, Spirochaetes, which was extremely rare in the modern human but enriched in ancient humans [33], was also enriched in the baboon gut (Fig. 1). *Lactobacillus* was the most abundant genus in the baboon gut but presented a low abundance in the human gut (Fig. 3c). Notably, the important human gut bacterium, *Akkermansia* [34], was not detected in the baboon gut. Therefore, *Akkermansia* was more human-specific and thus absent in the baboons in our study, no *Akkermansia* has been reported in NHPs [35, 36].

## Conclusion

To our knowledge, this study is the first to investigate microbial compositions in cohabitating baboons across different body sites. Our results showed that baboons have a unique microbiome compared to humans. The microbial diversity of the baboon vagina was much higher than that of humans. *Lactobacillus* was not so predominant in baboon vagina as in the human vagina but was the most abundant genus in the baboon gut. The microbial compositions in the baboon reproductive tract and gut were similar. Oral cavity, vagina and gut in group living baboons shared more bacterial ASVs than humans. The significantly positive correlations of those shared ASVs between multiple body sites in this group of baboons combined with highly social characteristics of baboons indicated a potential bacterial exchange throughout the body. We reported that the probably transmitted bacteria across body sites tend to be bacteria known to be beneficial in humans, which may suggest that some modern human populations, due to changed social behaviors, may have lost an important source of beneficial microbiota with consequences for human health.

## Materials and methods

### Controls manage the risk of contamination during wet-lab processing and sterile surgical procedure manage the risk of contamination at sampling

To avoid contamination risks, we strictly controlled the sampling process, DNA extraction, PCR and sequencing. All DNA extractions strictly followed the aseptic operation process under a clean bench. We also have DNA extraction negative control (from the DNA extraction to the sequencing process), sequencing blank control (clean water for sequencing), and sequencing positive control (mock community, *E.coli*). The detailed information is included in the supplementary material.

### Sample collection

Samples were collected from 16 captive baboons (*Papio hamadryas,* 13 females, 3 males) housed at the Copenhagen Zoo, Denmark. Seven different sites were sampled (Fig. 1) and all sample information is listed in Table S5. Animals were anesthetized for a full medical evaluation and physical examination. Non-invasive samples (vagina, nose, oral, oropharynx, and gut) were collected in a sheltered housing facility using sterile polyester swabs (cat no. 300263, Deltalab, Spain). Following thorough medical evaluations, eight of the animals were euthanized by a licensed veterinarian and a thorough postmortem examination was conducted in a separate necropsy room. Carcasses were opened ventrally to expose the organs, and all invasive sampling was performed sequentially from cranial to caudal. A new sterile scalpel was used for each organ; new gloves were donned and new surgical utensils were used for each of the carcasses. Animals were euthanized for reasons unrelated to this project.

### DNA extraction and 16S rRNA gene amplicon sequencing

Genomic DNA was extracted from swab samples with the PowerLyzer® PowerSoil® DNA Isolation Kit (MO-BIO Laboratories, Inc., Carlsberg, CA, USA), and 50 μL of elution buffer was used for each sample. All operations were performed under aseptic conditions. Extracted DNA was stored at − 20 °C. Sterilized PBS solution and Molecular grade water (Sigma-Aldrich, United States) were used as DNA extraction and DNA amplification negative control, whereas mock community and only *Escherichia.coli* strain were included in all the following steps as a positive control. The 16S rRNA gene hypervariable V3-V4 region was amplified with 2 μL template DNA, using 0.25 μL Phusion high-fidelity (HF) DNA Polymerase (Thermo Fisher Scientific, Waltham, MA, USA), 5 μL 5 × Phusion buffer HF, 0.5 μL 10 mM dNTPs, 1 μL 10 μM of each primer (the modified broad primers 341F (5′-CCTAYGGGRBGCASCAG-3′) and Uni806R (5′-GGACTACNNGGGTATCTAAT-3′) [37] in a 25 μL PCR reaction volume. The first PCR program included 30 s at 98 °C, 30 cycles of 5 s at 98 °C, 15 s at 56 °C, and 72 °C for 10 s, and then 5 min at 72 °C. In the second PCR, sequencing primers and adaptors were attached to the amplicon library following the first PCR conditions with only 15 cycles. The size of the PCR product (≈466 bp) was evaluated using gel electrophoresis. The amplicon products were purified by use of Agencourt AMPure XP beads (Beckman Coulter Genomics, MA, USA) with the 96-well magnet stand, normalized by the SequalPrepTM Normalization Plate (96) kit (Invitrogen Ltd., Paisley, UK), pooled in equimolar concentrations and concentrated using the DNA Clean & Concentrator™-5 Kit (Zymo Research, Irvine, CA, USA). Sequencing of the amplicon library was performed on the Illumina MiSeq System with MiSeq reagent kit v2 (Illumina Inc., CA, USA), including 5.0% PhiX as an internal control.

### Bioinformatics analysis and statistical analysis

The raw fastq files were demultiplexed using the Miseq Controller Software. Primers and diversity spacers were removed from fastq files using “Cutadapt” [38]. The data trimming and feature classification were done using QIIME 2 Core 2017.12 distribution microbiota analysis platform [39]. Paired-end sequences were merged by vsearch plugin [40] and then followed by filtering with the quality-filter plugin [41], both with default settings. Deblur plugin was then used to denoise the sequences with a trim length of 400 bp based on quality score plots [42]. Sequence alignments were generated using MAFFT and the aligned sequences were masked by MASK plugin [43]. FastTree and midpoint-root built-in phylogeny plugin were used to create a rooted phylogenetic tree [44]. Pre-fitted sklearn-based taxonomy classifier (https://github.com/QIIME2/q2-feature-classifier) was used to blast representative sequences against silva 132 database for taxonomic classification of features [45]. Rarefaction curves were plotted by alpha_rarefaction.py workflow in QIIME 2.

A histogram of average unweighted UniFrac distances between each sample and all the rest of the samples were plotted to confirm that PCR and Sequencing controls differed from our samples (supplementary material). The DNA amplification negative control with only 63 reads was filtered. The histogram showed that the three controls (DNA extraction blank control, *E.coli* strain and Mock control) were far away from the real samples, which meant controls were different from the real samples. The rarefaction curves (supplementary material) demonstrated the observed richness in a given count of sequences. Observed richness curves reached asymptotes after 4000 reads for most samples. With an average of 15,247 clean sequences per sample, sufficient sequences for all 170 samples were generated to characterize the microbial community in the seven body habitats.

The open-source statistical program “R” was used for data treatment and statistical analysis [46], predominantly the *R*-package “phyloseq” [47]. Alpha diversity between the groups was tested by analysis of variance using the function “ano”. If significant differences between the groups were present, multiple comparisons with the function “TukeyHSD” were performed in a pairwise manner between all groups (all two functions from *R*-package “stats”). Bray Curtis distance was used to explain differences among microbial communities and the dissimilarity was examined by permutational multivariate analysis of variance (PERMANOVA, “vegan” function “adonis”) [48]. *R* Function “pairwise.adonis” [49] was used for multiple comparisons and Bonferroni correction was used to account for multiple comparisons. Group Divergence was quantified as the average dissimilarity of each sample from the group mean by using the function “divergence” from R-package “microbiome” [50]. Venn diagram was plotted by *R* function “VennCounts” and “VennDiagram” in *R* package “VennDiagram” [51]. *R* function “rcorr” was used to compute the Spearman correlation analysis and the significance levels [52]. The correlation matrix and the significance test were visualized by *R* function “corrplot” [53]. Pie chart, donut chart, violin plot, box plot, heatmap, bar chart and circle bar chart were plotted using ggplot2 [54].

## Supplementary information

**Additional file 1. Supplementary table**: sample overview; statistical test tables.

**Additional file 2. Supplementary material**: controls manage risk of contamination during wet-lab processing and sterile surgical procedure manage risk of contamination at sampling.

**Additional file 3.**

## Data Availability

The dataset analyzed during the current study will be available, upon publication, in the Sequence Read Archive (SRA) repository, http://www.ncbi.nlm.nih.gov/bioproject/464237. All the scripts used in this study can also be found at, http://mibi.galaxy.bio.ku.dk/XuanJi_Baboon_Microbiome/scripts/.

## Abbreviations

NHPs: Nonhuman primates
ASV: Amplicon sequence variant
PERMANOVA: Permutational Multivariate Analysis of Variance
NMDS: Non-metric multidimensional scaling
MAFFT: Multiple sequence alignment program
PCR: Polymerase chain reaction
