# Supplementary figures and images for: The microbiome of captive hamadryas baboons
Source: Anim Microbiome. 2020 Jul 16;2:25. doi: 10.1186/s42523-020-00040-w (PMC7807707; doi:10.1186/s42523-020-00040-w)

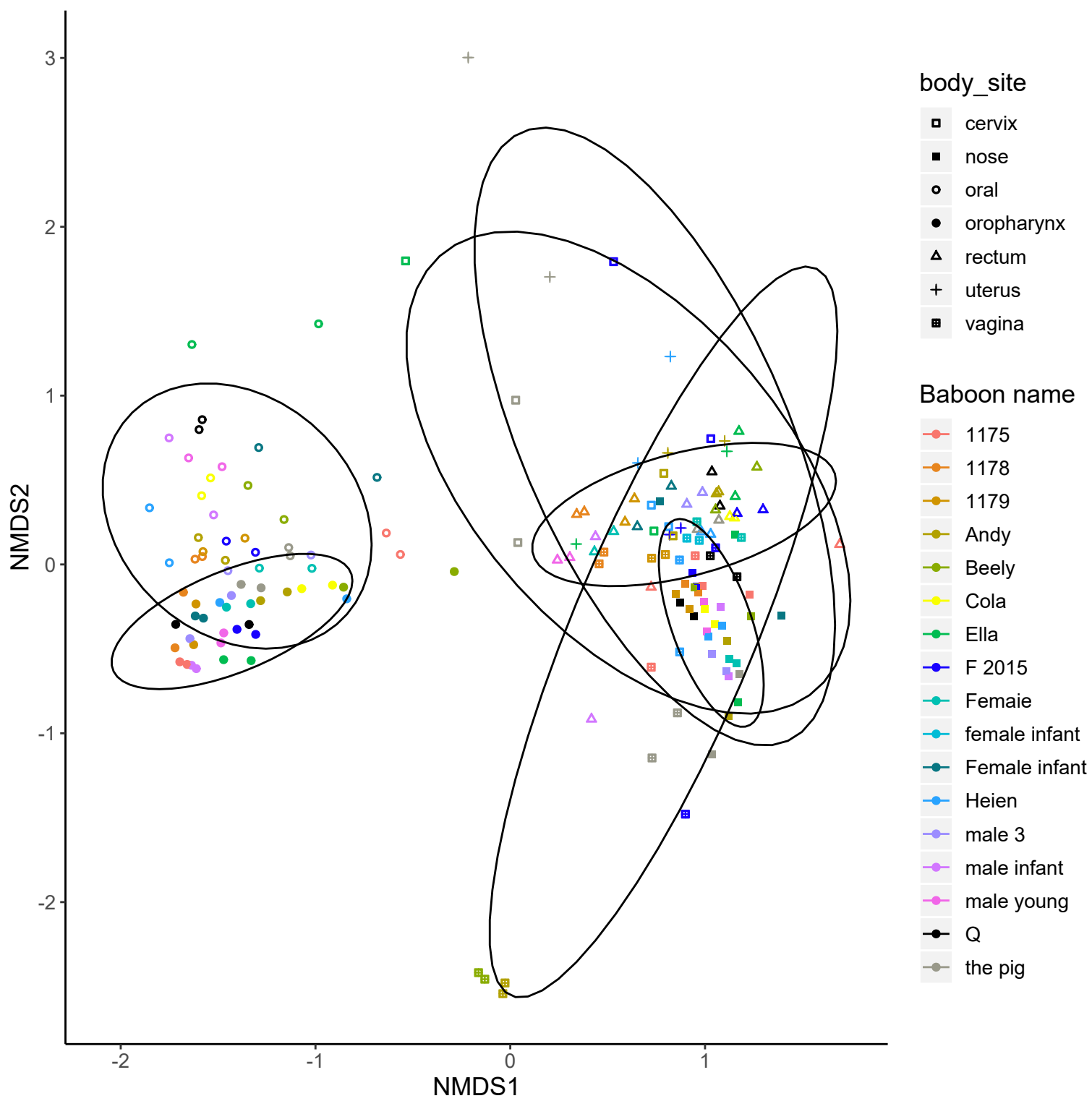

Supplement: Supplementary file 3 — Additional file 3. [file 42523_2020_40_MOESM3_ESM.pdf]
